# Lower Limb Joint Torque Prediction Using Long Short-Term Memory Network and Gaussian Process Regression †

**DOI:** 10.3390/s23239576

**Published:** 2023-12-02

**Authors:** Mengsi Wang, Zhenlei Chen, Haoran Zhan, Jiyu Zhang, Xinglong Wu, Dan Jiang, Qing Guo

**Affiliations:** 1 School of Aeronautics and Astronautics, University of Electronic Science and Technology of China, Chengdu 611731, China; ms_wang@std.uestc.edu.cn (M.W.); hr.zhan@std.uestc.edu.cn (H.Z.); w947115904@163.com (X.W.); 2 Aircraft Swarm Intelligent Sensing and Cooperative Control Key Laboratory of Sichuan Province, Chengdu 611731, China; 3 School of Automation Engineering, University of Electronic Science and Technology of China, Chengdu 611731, China; zhenlei_chen@std.uestc.edu.cn; 4 School of Instrumentation Science and Engineering, Harbin Institute of Technology, Harbin 150001, China; 19b901013@stu.hit.edu.cn; 5 School of Mechanical and Electrical Engineering, University of Electronic Science and Technology of China, Chengdu 611731, China; jdan2002@uestc.edu.cn

**Keywords:** joint torque, electromyography signals, machine learning, long short-term memory, Gaussian process regression

## Abstract

The accurate prediction of joint torque is required in various applications. Some traditional methods, such as the inverse dynamics model and the electromyography (EMG)-driven neuromusculoskeletal (NMS) model, depend on ground reaction force (GRF) measurements and involve complex optimization solution processes, respectively. Recently, machine learning methods have been popularly used to predict joint torque with surface electromyography (sEMG) signals and kinematic information as inputs. This study aims to predict lower limb joint torque in the sagittal plane during walking, using a long short-term memory (LSTM) model and Gaussian process regression (GPR) model, respectively, with seven characteristics extracted from the sEMG signals of five muscles and three joint angles as inputs. The majority of the normalized root mean squared error (NRMSE) values in both models are below 15%, most Pearson correlation coefficient (*R*) values exceed 0.85, and most decisive factor ($R^{2}$) values surpass 0.75. These results indicate that the joint prediction of torque is feasible using machine learning methods with sEMG signals and joint angles as inputs.

## 1. Introduction

The real-time and accurate prediction of lower limb joint torque has important research significance in many fields. In sports rehabilitation, it serves as a foundation for the understanding of changes in people’s muscle strength, and it enables doctors to guide the process of rehabilitation training effectively [1,2]. In human–machine interaction systems, it forms the basis for machines to discern human motor intentions and adjust assistance strategies promptly [3,4,5]. Unfortunately, the use of mechanical torque sensors to measure subjects’ active joint torque is challenging because the sensed torque signals include undesired torque components, such as gravity torque, friction torque, and inertial torque, which need to be eliminated using complicated processing algorithms [6]. Currently, three methods are employed to compute joint torque, namely the inverse dynamics model, EMG-driven NMS model, and machine learning model.

The inverse dynamics model [7,8] is considered a standard method that requires a complex experimental environment and equipment. Two common approaches to developing an inverse dynamics model are the Newton–Euler equations and Lagrange equations. Both methods rely on GRF measurements, which are typically only available in laboratory settings.

The EMG-driven NMS model builds upon the muscle–tendon model, which can be traced back to the Hill-type muscle model proposed in 1938 [9]. At present, the improved model proposed by Buchanan and Lloyd is the most widely used [10,11]. However, the EMG-driven NMS model faces three major challenges. Firstly, measuring individual physiological parameters in vivo is difficult. Secondly, the model requires input from all muscles involved in joint motion. Thirdly, the iterative optimization process for model parameters is tedious.

In recent years, machine learning models have gained popularity in many research fields due to their ability to learn from large amounts of data without relying on explicit equations [12,13,14]. As sEMG signals exhibit smaller time delays and a higher signal-to-noise ratio (SNR), it may be an ideal method to use sEMG signals to estimate joint torque [6]. Meanwile, user-friendly wireless sEMG sensors make it possible to accurately measure sEMG signals during human movement. Therefore, various non-model-based machine learning regression methods, such as regression trees (RT) [15,16,17], support vector machines (SVM) [18,19,20], neural networks (NN) [21,22,23], and Gaussian process regression (GPR) [24,25,26], have been applied to predict joint torque using sEMG signals and motion information (joint angles, angular velocity, angular acceleration, etc.), which can be measured with portable devices such as sEMG sensors integrated with inertial measurement units (IMUs).

Among neural network models, the long short-term memory (LSTM) model has shown excellent performance in time series prediction tasks, as it effectively captures and remembers long-term dependencies through its gating unit design. The use of an LSTM model to predict joint torque with sEMG signals and motion information is a suitable choice, as all these variables are time series data related to human movement. Previous studies have demonstrated the effectiveness of using LSTM models for torque estimation. Siu et al. [27] found that an LSTM model used to estimate ankle torque with sEMG signals and accelerometry as inputs outperformed other methods, such as dense feedforward neural networks (FNN), convolutional neural networks (CNNs) [28,29], and neural ordinary differential equations (ODEs). Zhang et al. [30] observed that an LSTM model could predict lower limb joint torque during various activities accurately, with a relatively low error, using sEMG signals and joint angles as inputs. Truong et al. [31] extracted several sEMG features to predict joint angles and joint torque using an LSTM model when squatting, picking up an object, and sitting–standing.

Unlike the point prediction in NNs, GPR can not only predict the value of the target variable but also estimate the uncertainty of the prediction. It provides reliability to the prediction by calculating the confidence interval. Yang et al. [32] estimated the joint torque with a GPR model using the GRF and foot motion from wearable smart shoes while walking at three different speeds. Most $R^{2}$ values in their experiment were higher than 0.8, indicating good predictive performance. Ullauri et al. [33] compared a GPR model and pneumatic artificial muscle (PAM) model with muscle activation calculated by measuring sEMG signals to predict elbow torque, and they found that GPR provided relatively more favorable predictions.

**Figure 1:**
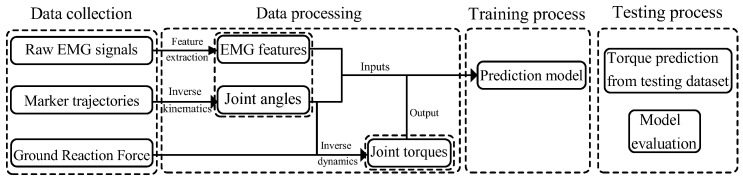
The overall workflow of this study. First, raw sEMG signals, marker trajectories, and ground reaction forces are collected; next, after being processed, the inputs (sEMG features and joint angles) and the output (joint torques) are obtained; then, parameters in the model are trained using data from the training set; finally, the performance of the models is evaluated using data from the testing set.

Based on previous research about joint torque estimation by BPNN learning [34], this study aims to predict the joint torque during walking using an LSTM model and GPR model with sEMG signals and joint angles as inputs. Figure 1 shows the workflow of this work. This study can be referenced for the prediction of joint torque when only sEMG signals and joint angles are available and the GRF cannot be measured. The main contributions of this work are as follows:

- The EMG signals, kinematics, and dynamics data are collected and processed during the normal walking of four subjects.
- An LSTM model and GPR model are built to predict torque using EMG signals and joint angles without GRF.

This article is structured as follows. Section 2 describes the methods of data collection and data processing, and the principles of the LSTM model and GPR model. Section 3 shows the results of the two models. Section 4 discusses the performance and limitations of the two models. Conclusions and future work are given in Section 5.

## 2. Materials and Methods

### 2.1. Data Collection

Four healthy young male subjects (mean ± STD, age ± 0.5 years, height ± 4.30 kg, weight ± 3.0, Table 1) were recruited to participate in the data collection experiment. All subjects were asked to perform 5 walking trials at a speed of 0.8 m/s on a 5-m flat surface with 7 embedded force platforms. sEMG data, force data, and motion capture data were collected simultaneously. The collected data were divided according to the gait cycle (from left foot toe-off to the subsequent left toe-off). Multiple data of 5 complete walking gait cycles for each subject were acquired.

#### 2.1.1. EMG Data Collection

**Figure 2:**
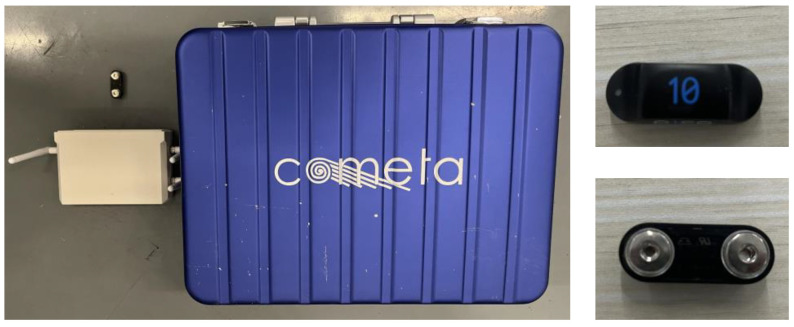
Pico EMG, Cometa Systems.

**Figure 3:**
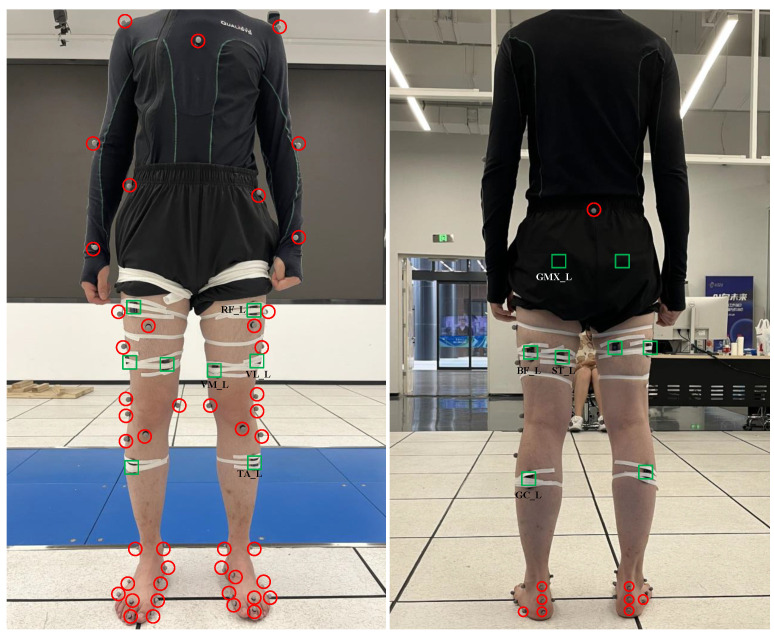
The locations where EMG sensors and reflective markers were placed on the subject’s body.

sEMG signals were collected using 16 wireless sEMG sensors (Pico EMG, Cometa Systems, Inc., Newburg, MO, USA, Figure 2) at 2000 Hz. For each subject, 16 sEMG signals were collected simultaneously from the gluteus maximus (GMX), rectus femoris (RF), vastus medialis (VM), vastus lateralis (VL), biceps femoris (BF), semitendinosus (ST), tibialis anterior (TA), and gastrocnemius (GC) of both the left and right legs. Figure 3 illustrates the locations where the EMG sensors were placed on the subject’s body. sEMG signals from GMX, RF, BF, TA, and GC from the left leg were used in this study.

#### 2.1.2. Force and Motion Data Collection

**Figure 4:**
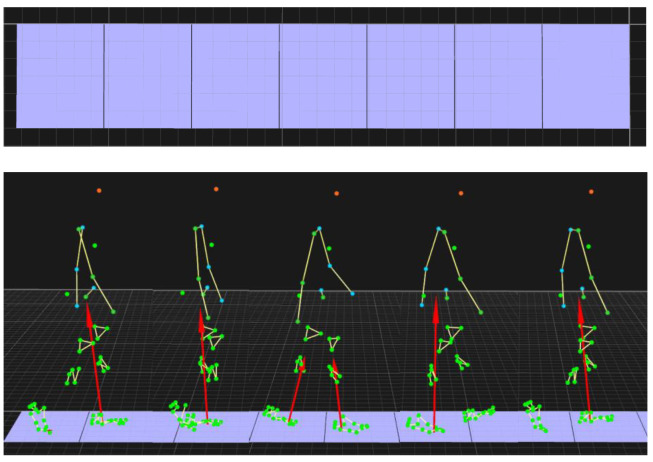
Qualisys 3D motion capture system.

Force and motion data were collected using a 3D motion capture system (Qualisys, Goteborg, Sweden, Figure 4) at 100 Hz. Subjects were asked to perform movements with their legs stepping on different force plates. A total of 51 reflective markers were fixed on the subjects to record motion trajectories (Figure 3). The GRF data were collected by 7 platforms and the marker trajectories were captured by 41 cameras.

The sEMG signals, joint angles, and joint torque of the left leg of each subject were studied in this work.

### 2.2. Data Processing

#### 2.2.1. sEMG Feature Extraction

The raw sEMG signals were band-pass filtered (20–450 Hz) by a 4th-order zero-lag Butterworth filter [35]. Then, 5 time domain features, namely the mean absolute value (MAV), root mean square (RMS), zero crossing (ZC), slope sign change (SSC), and waveform length (WL) [36,37,38], and 2 frequency domain features, namely the mean frequency (MNF) and median frequency (MDF) [39], were extracted. The time domain features were extracted using a 150-ms (300 points) movable overlapped window, and the frequency domain features were extracted using a 128-ms (256 points) movable overlapped window.

- MAV:
  (1) $$MAV=\frac{1}{N}\sum_{i=1}^{N}\left|X_{i}\right|,$$
  where *X* represents the EMG signal in a movable window and *N* represents the window length.
- RMS:
  (2) $$RMS=\sqrt{\frac{1}{N}\sum_{i=1}^{N}X_{i}^{2}}.$$
- ZC:
  (3) $$ZC=\sum_{i=1}^{N-1}sgn\left(X_{i}\cdot X_{i+1}\right)\cap \left|X_{i}-X_{i+1}\right|\geq 0,$$
  where
  $$sgn\left(x\right)=\left\{\begin{array}{cc} 1, & \mathrm{if}\;x\geq 0\\ 0, & \mathrm{otherwise}. \end{array}\right.$$
- SSC:
  (4) $$SSC=\sum_{i=2}^{N-1}sgn\left(\left(X_{i}-X_{i-1}\right)\cdot \left(X_{i}-X_{i+1}\right)\right).$$
- WL:
  (5) $$WL=\sum_{i=1}^{N-1}\left|X_{i}-X_{i+1}\right|.$$
- MNF:
  (6) $$MNF=\frac{\sum_{j=1}^{M}f_{j}P_{j}}{\sum_{j=1}^{M}P_{j}},$$
  where $f_{j}$ is the *j*th frequency component, $P_{j}$ is the power spectrum at $f_{j}$, and *M* is the total number of frequency components.
- MDF:
  (7) $$\sum_{j=1}^{MDF}P_{j}=\sum_{j=MDF}^{M}P_{j}=\frac{1}{2}\sum_{j=1}^{M}P_{j}.$$

EMG signals were downsampled to 100 Hz after feature extraction to correspond to force and motion data.

#### 2.2.2. Inverse Kinematics

Joint angles were calculated by the inverse kinematics toolbox in OpenSim 4.4 (an open-source software system for biomechanical modeling, simulation, and analysis, SimTK, Stanford, CA, USA). The Gait2392_Simbody model was chosen for the research in this study. First, for each subject, a scaled model was built to match the markers on his body best. Then, the joint angles were obtained by optimizing the error between the virtual marker positions on the scaled model and the real marker positions from experiments. The detailed objective function is as follows:

(8) $$\underset{q}{min}\left[\sum_{i\in markers}\omega_{i}\|{x_{i}^{exp}-x_{i}\left(q\right)\|}^{2}\right],$$

where $\omega_{i}$ is the weight of the marker *i*, q is the joint angle vector being solved for, $x_{i}^{exp}$ is the experimental position of marker *i*, $x_{i}\left(q\right)$ is the virtual position of marker *i* at given joint angle vector q, and ∥·∥ represents the standard Euclidean norm.

#### 2.2.3. Inverse Dynamics

Joint torque was calculated by the inverse dynamics toolbox in OpenSim 4.4 after joint angles were obtained. The classical equations of motion can be written as follows:

(9) $$M\left(q\right)\ddot{q}+C\left(q,\dot{q}\right)+G\left(q\right)=\tau ,$$

where q, $\dot{q}$, $\ddot{q}$ are angles, angular velocities, and angular accelerations, respectively; M(q) is the mass matrix; $C(q,\dot{q})$ is the vector of Coriolis and centrifugal torque; G(q) is the vector of gravitational torque; and τ is the vector of the unknown generalized torque.

The features of sEMG signals, the joint angles, and the joint torque were normalized by the maximum value of each channel. The input signal was 38-dimensional (7 features of 5 sEMG signals and 3 joint angles).

### 2.3. LSTM Neural Network Model

**Figure 5:**
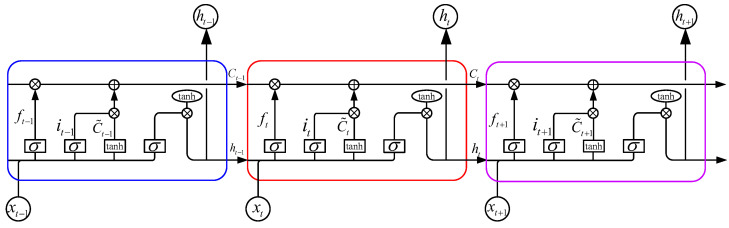
The structure of a typical LSTM unit.

LSTM is a type of recurrent neural network (RNN) that is designed to overcome the limitations of traditional RNNs in capturing long-term dependencies in sequential data. LSTM units, which are the building blocks of an LSTM network, are more complex than traditional RNN units. A typical LSTM unit is composed of a cell state, an input gate, an output gate, and a forget gate [40,41], as Figure 5 shows. These components work together to process sequential data and maintain information over long periods.

The specific forward propagation formulas of an LSTM unit are as follows. First, the forget gate decides which information to discard in the last cell state,

(10) $$f_{t}=\sigma \left(W_{f}\cdot x_{t}+U_{f}\cdot h_{t-1}+b_{f}\right),$$

where $f_{t}\in {\left(0,1\right)}^{h}$ represents the forget gate’s activation vector, $x_{t}\in R^{d}$ represents the input with *d* features at time *t*, $h_{t-1}\in R^{h}$ represents the output at time t−1, $W_{f}\in R^{h\times d}$ and $U_{f}\in R^{h\times h}$ represent the weight matrix, $b_{f}\in R^{h}$ represents the bias vector, and σ represents the sigmoid activation function. Second, the input gate determines which information to keep,

(11) $$i_{t}=\sigma \left(W_{i}\cdot x_{t}+U_{i}\cdot h_{t-1}+b_{i}\right),$$

(12) $${\tilde{c}}_{t}=tanh\left(W_{c}\cdot x_{t}+U_{c}\cdot h_{t-1}+b_{c}\right),$$

where $i_{t}\in {\left(0,1\right)}^{h}$ represents the update gate’s activation vector, ${\tilde{c}}_{t}\in {\left(-1,1\right)}^{h}$ stands for the cell input activation vector, $W_{i},W_{c}\in R^{h\times d},U_{i},U_{c}\in R^{h\times h}$ represents the weight matrix, $b_{i},b_{c}\in R^{h}$ represents the bias vector, and tanh represents the hyperbolic tangent activation function. Then, the cell state is updated from time t−1 to time *t*,

(13) $$c_{t}=f_{t}⊙c_{t-1}+i_{t}⊙{\tilde{c}}_{t},$$

where $c_{t}\in R^{h}$ is the cell state vector at time *t*, and $c_{t-1}$ is the cell state vector at time t−1. Finally, the output is obtained,

(14) $$o_{t}=\sigma \left(W_{o}\cdot x_{t}+U_{o}\cdot h_{t-1}+b_{o}\right),$$

(15) $$h_{t}=o_{t}⊙tanh\left(c_{t}\right),$$

where $o_{t}\in {\left(0,1\right)}^{h}$ represents the output gate’s activation vector; $h_{t}\in {\left(-1,1\right)}^{h}$ is the hidden state vector, also known as the output vector of an LSTM unit; $W_{o}\in R^{h\times d}$ and $U_{o}\in R^{h\times h}$ represent the weight matrices; $b_{o}\in R^{h}$ represents the bias vector; and the operator ⊙ represents the Hadamard product. The initial values are $c_{0}=0$ and $h_{0}=0$, and the parameters *W*, *U*, and *b* need to be learned during the training process.

**Figure 6:**
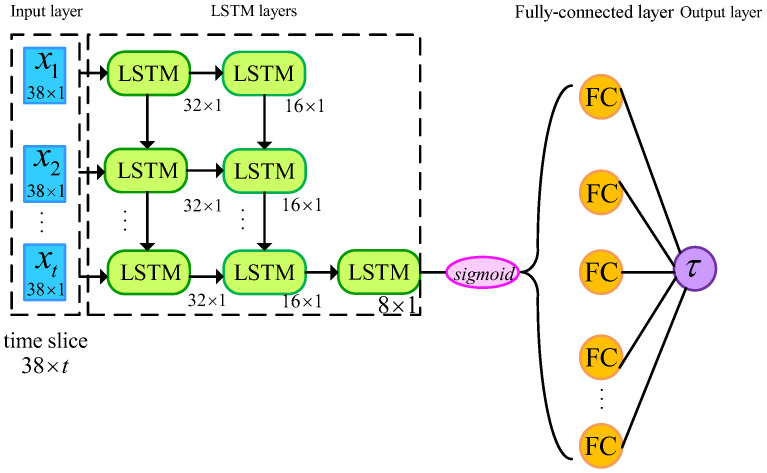
The structure of the LSTM network model.

In this study, a network model with 3 LSTM layers and a fully connected (FC) layer was built using the deep learning toolbox of Matlab 2021b. The architecture of the model is shown in Figure 6. Each LSTM layer consisted of 32, 16, and 8 hidden neuron units, respectively, and the output layer had 1 neuron. The output mode of the first and second LSTM layers was set to ‘sequence’, and the output mode of the third LSTM layer was set to ‘last’. A sigmoid layer was included between the third LSTM layer and the fully connected layer. The solver was set to be ‘adam’ and the training was performed for 500 epochs. To prevent the gradients from exploding during training, a gradient threshold of 1 was set. The initial learning rate was set to 0.005. After 125 epochs, the learning rate was dropped by multiplying it with a factor of 0.2. The format of the input data was transformed to time slices (sample number × features × time step). The time step was set to be 5, which was equivalent to 50 ms. The model was trained to predict the output at the next time step, which was 10 ms ahead of real time. For each joint of each subject, a model was trained individually. In this study, 12 different LSTM models were obtained.

### 2.4. GPR Model

The Gaussian process (GP) is a non-parametric learning method that has advantages over parametric methods when given a small training set [42]. Unlike point prediction in NNs, GP can quantify the uncertainty of the point prediction, which is favorable to decrease risks in decision making. In the regression model, decision makers can forecast the possible outcomes with an explicit probability when provided a 95% confidence interval around the prediction [43].

Any finite subset of a set of random variables {f(x)|x∈X} that follows a Gaussian distribution is known as a Gaussian process [44]. To specify a GP, the mean function m(x) and the covariance function $k(x,x^{{}^{\prime }})$ should be defined, which contain a series of hyperparameters θ [45,46]. Then, the GP can be written as

(16) $$f\left(x\right)∼\mathrm{GP}(m\left(x\right),k\left(x,x^{{}^{\prime }}\right)).$$

Given a training data set $D\left(X,y\right)=\{\left(x_{1},y_{1}\right),\left(x_{2},y_{2}\right),\ldots ,\left(x_{N},y_{N}\right)\}$ of N pairs of vectorial input $x_{i}$ and noisy scalar output $y_{i}$, $y_{i}$ is obtained by the latent function $f(x_{i})$ with Gaussian noise

(17) $$y_{i}=f\left(x_{i}\right)+\epsilon_{i},$$

where $f\left(x\right)∼\mathrm{GP}(0,k\left(x,x^{{}^{\prime }}\right))$,$\epsilon_{i}∼N\left(0,\sigma^{2}\right)$. We have

(18) f∼N(0,K(X,X)),

(19) $$y∼N(0,K\left(X,X\right)+\sigma^{2}I).$$

For a testing data set $D=(X_{*},y_{*})$ of $N_{*}$ points, the joint distribution $p(y,f_{*})$ can be obtained as

(20) $$\left[\begin{array}{c} y\\ f_{*} \end{array}\right]∼N\left(0,\left[\begin{array}{cc} K_{NN}+\sigma^{2}I & K_{NN^{*}}\\ K_{N^{*}N} & K_{N^{*}N^{*}} \end{array}\right]\right),$$

where $K_{NN^{*}}$ denotes the $N\times N_{*}$ covariance matrix of the training points and the testing points. $K_{NN}$, $K_{N^{*}N}$ and $K_{N^{*}N^{*}}$ are similar. The Gaussian predictive distribution $p(f_{*}|y)$ can be obtained from the joint distribution $p(y,f_{*})$ as

(21) $$f_{*}|y∼N\left({\bar{f}}_{*},cov\left(f_{*}\right)\right),$$

where

(22) $${\bar{f}}_{*}=K_{N^{*}N}{\left[K_{NN}+\sigma^{2}I\right]}^{-1}y,$$

(23) $$cov\left(f_{*}\right)=K_{N^{*}N^{*}}-K_{N^{*}N}{\left[K_{NN}+\sigma^{2}I\right]}^{-1}K_{NN^{*}}.$$

The training process of GP is actually to learn the hyperparameters ($\theta ,\sigma^{2}$) by maximizing the log-marginal likelihood logp(y)=∫p(y|f)p(f)df, namely

(24) $$logp\left(y\right)=-\frac{1}{2}y^{⊤}{\left(K+\sigma^{2}I\right)}^{-1}y-\frac{1}{2}log\left|K+\sigma^{2}I\right|-\frac{n}{2}log2\pi ,$$

where K denotes K(X,X) in (9).

In this study, the GPR model was implemented using the regression learner in Matlab 2021b. The goal was to predict the torque at the current time point based on input feature data from the previous time point, with a prediction horizon of 10 ms ahead of real time. After evaluating the performance of 4 kinds of kernel functions (rational quadratic, squared exponential, matern5/2, exponential), the exponential kernel function was finally selected. The basis function was set to be constant and the standardization was set to be true. For each joint of each subject, a separate GPR model was trained, resulting in a total of 12 distinct GPR models in this study.

### 2.5. Evaluation Protocol

For each subject, 5 complete gait cycles were acquired. Out of the 5 gait cycles, 4 cycles were used as the training set to train the models, while the remaining cycle was used as the testing set to evaluate the performance of the models.

The NRMSE is as follows:

(25) $$NRMSE=\frac{RMSE}{y_{max}-y_{min}}\times 100\%,$$

where $RMSE=\sqrt{\frac{1}{N}\sum_{i=1}^{N}{\left(y_{i}-{\hat{y}}_{i}\right)}^{2}}$ represents the root mean squared error, the Pearson correlation coefficient *R*,

(26) $$R=\frac{\sum_{}^{}\left(y_{i}-{\bar{y}}_{i}\right)\left({\hat{y}}_{i}-{\bar{\hat{y}}}_{i}\right)}{\sqrt{\sum_{}^{}{\left(y_{i}-{\bar{y}}_{i}\right)}^{2}{\left({\hat{y}}_{i}-{\bar{\hat{y}}}_{i}\right)}^{2}}}$$

and the decisive factor $R^{2}$,

(27) $$R^{2}=1-\frac{SSE}{SST},$$

where $SSE=\sum_{i=1}^{N}{\left(y_{i}-{\hat{y}}_{i}\right)}^{2}$, $\sum_{i=1}^{N}{\left(y_{i}-{\bar{y}}_{i}\right)}^{2}$, are all used to evaluate the model performance. A lower NRMSE and higher *R* and $R^{2}$ close to 1 indicate better regression performance.

## 3. Results

### 3.1. The Result of the LSTM Model

Table 2 shows the performance of the LSTM model in predicting joint torque for all subjects. For the hip joints of four subjects, the NRMSE values are less than 15%, the *R* values are more than 0.92, and the $R^{2}$ values are more than 0.79. For the knee joint, the NRMSE values are more than 17%, the *R* values are more than 0.83, and the $R^{2}$ values are more than 0.62. For the ankle joint, the NRMSE values are less than 8%, the *R* values are more than 0.95, and the $R^{2}$ values are more than 0.90.

**Figure 7:**
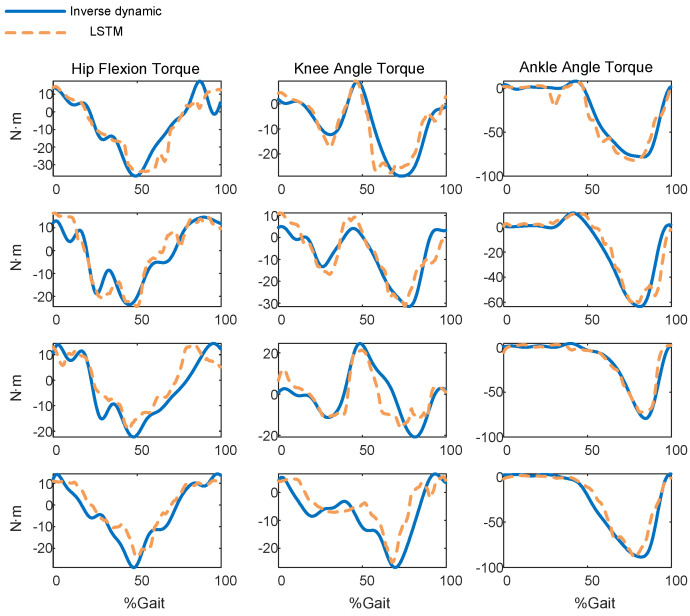
The predicted torque using LSTM network model.

Certain differences exist between different people. The best hip prediction occurs on subject 4, of which the NRMSE value is 11.2691, the *R* value is 0.9469, and the $R^{2}$ value is 0.8512. The best knee prediction occurs on subject 1, of which the NRMSE value is 12.3561, the *R* value is 0.9102, and the $R^{2}$ value is 0.8207. The best ankle prediction occurs on subject 3, of which the NRMSE value is 7.6790, the *R* value is 0.9837, and the $R^{2}$ value is 0.9646. Figure 7 displays the real torque and the predicted torque of the LSTM model.

### 3.2. The Result of the GPR Model

Table 3 presents the performance of the GPR model in predicting the joint torque for all subjects. For the hip joints of four subjects, the NRMSE values are less than 17%, the *R* values are more than 0.91, and the $R^{2}$ values are more than 0.75. For the knee joint, the NRMSE values are less than 18%, the *R* values are more than 0.85, and the $R^{2}$ values are more than 0.56. For the ankle joint, the NRMSE values are less than 8%, the *R* values are more than 0.94, and the $R^{2}$ values are more than 0.81.

**Figure 8:**
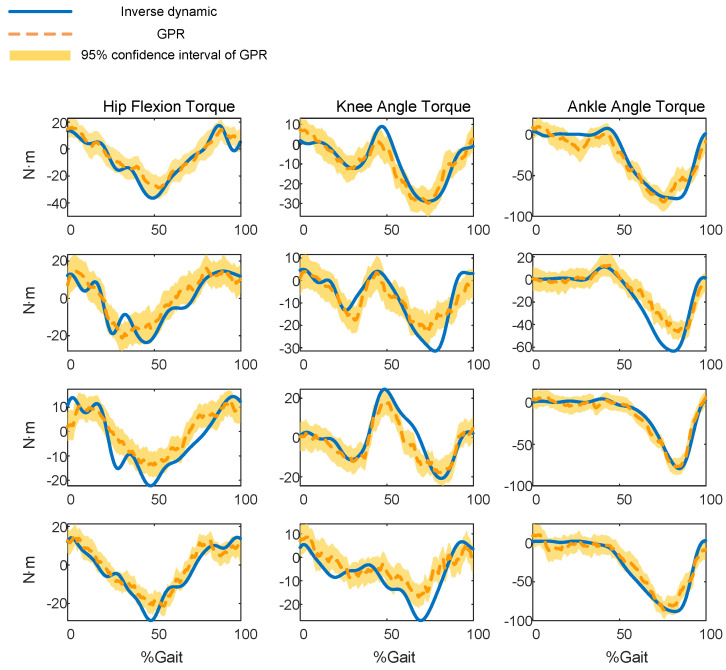
The predicted torque using GPR model.

The best hip prediction occurs on subject 1, of which the NRMSE value is 8.6833, the *R* value is 0.9661, and the $R^{2}$ value is 0.9070. The best knee prediction occurs on subject 3, of which the NRMSE value is 11.4735, the *R* value is 0.9328, and the $R^{2}$ value is 0.7999. The best ankle prediction occurs on subject 3, of which the NRMSE value is 6.2121, the *R* value is 0.9801, and the $R^{2}$ value is 0.9590. Figure 8 shows the real torque and the predicted torque of the GP model, with shaded areas representing the 95% confidence intervals of the predicted torque.

## 4. Discussion

In this study, an LSTM model and GPR model were used to predict joint torque in the sagittal plane during walking, using sEMG signals and joint angles as inputs. The relevant regression performance (NRMSE, *R*, $R^{2}$) indicated that the predicted torque was in good agreement with the torque calculated by inverse dynamics. In the LSTM model, the average regression performance for the ankle (NRMSE: 6.5850%, *R*: 0.9711, $R^{2}$: 0.9361), hip (NRMSE: 12.5728%, *R*: 0.9324, $R^{2}$: 0.8336), and knee (NRMSE: 14.4465%, *R*: 0.8763, $R^{2}$: 0.7560) decreased in sequence. In [27], Siu et al. took sEMG signals and accelerations as inputs, using an LSTM model to predict the ankle torque (average *R* of five subjects: 0.84) accurately. However, only the maximum value and area for each window of the sEMG signals were extracted. In our study, seven features of sEMG signals were extracted, and, in comparison, the average *R* was improved. In the GPR model, the average regression performance for the ankle (NRMSE: 6.4766%, *R*: 0.9636, $R^{2}$: 0.9018), hip (NRMSE: 12.3216%, *R*: 0.9391, $R^{2}$: 0.8371), and knee (NRMSE: 14.3591%, *R*: 0.8980, $R^{2}$: 0.7335) also decreased in order. In [32], Yang et al. used a GP model to predict joint torque with plantar pressure, orientations, and angular velocities as inputs. In the case of walking at the speed of 0.8 m/s, the prediction of ankle (average $R^{2}$: 0.8905), knee (average $R^{2}$: 0.7250), and hip (average $R^{2}$: 0.7390) torque was precise. However, it is inconvenient to measure plantar pressure with smart shoes. In our study, the measurement of the sEMG signal was more user-friendly. Portable wireless sEMG sensors integrated with IMUs made it possible to predict the joint torque in real time.

Although the low NRMSE and the high *R* and $R^{2}$ in both the LSTM model and GPR model indicate that the joint torque has a relatively strong correlation and high consistency with sEMG signals and joint angles, there still exist some limitations. The performance of the hip and knee torque is not as good as that of the ankle torque. In [30], Zhang et al. also used an LSTM model to predict joint torque, with good performance. Their study indicated that the best prediction performance during walking was for the hip, followed by the ankle and finally the knee. Our result was not exactly consistent with this. We suppose that the prediction performance can be essentially attributed to the complexity of the output curve to be predicted, including the number of data points, number of peaks, and data volatility. The more complex the output curve, the more challenging it is to accurately predict. Therefore, one solution to fundamentally improve the prediction performance is to add more valid EMG signals.

There are certain differences in the physiological and movement characteristics of different subjects, reflected in the data and results. In the LSTM model, the ability to estimate the knee joint torque of subject 1 was better than for subject 4. In the GP model, the ability to estimate the hip joint torque of subject 1 was better than for subject 3. For a specific subject, the regression performance would be improved if the adjustable parameters in the model (number of network layers, number of neurons, etc.) were individually adjusted.

Additionally, the EMG signal itself lacks robustness. In this research, the measurement of sEMG signals posed a challenge. The absolute amplitude of the sEMG signal itself has little reference significance. Factors such as the sensor itself, where the sensor is attached, and the tightness of the sensor attachment all affect the quality of the sEMG signal. To ensure consistency and reliability, all data must be collected in the same experiment, minimizing variations caused by these factors.

## 5. Conclusions and Future Work

In this study, the hip, knee, and ankle torque were predicted during walking in four subjects by an LSTM model and GPR model. The results indicate that both the LSTM and GPR models performed well in predicting the joint torque given the sEMG signals and joint angles as inputs, with low NRMSE values and high *R* and $R^{2}$. For all joints of each model, the average NRMSE values were all less than 15%, the *R* were all more than 0.87, and the $R^{2}$ were all more than 0.73. When using machine learning models like LSTM and GPR, on the one hand, there is no need to build dynamic models or measure the GRF compared to inverse dynamic models. On the other hand, the complex optimization process associated with NMS models is not necessary. The proposed models have potential application in various fields, including exoskeleton rehabilitation systems, exoskeleton assistance systems, and sports training. These models can provide accurate and real-time estimates of joint torque during walking when force plates are limited or unavailable.

In the future, our research group will conduct more in-depth research considering the following four aspects. Firstly, the diversity of the subjects should be increased. A larger data set including more subjects with different body types and different ages should be built. Additionally, studying subjects with lower limb disabilities has broader significance. Secondly, expanding the diversity of movements beyond walking would be beneficial. Including activities such as running, jumping, and cycling in daily life will provide a broader perspective on joint torque prediction across various dynamic movements. Thirdly, investigating inter-movement and inter-subject predictions is a more challenging task. It would involve using the current model and data set to predict joint torque for new movements and individuals. This research direction would contribute to a more comprehensive understanding of the generalizability and adaptability of the proposed model. Lastly, exploring torque prediction over a period of time, rather than at a single point in time, would be a valuable future direction.

## Figures and Tables

**Table 1:** The age, height, and weight of the subjects.

Subject	Age (Years)	Height (cm)	Weight (kg)
Subject 1	23.0	168.5	67.70
Subject 2	23.0	172.0	57.20
Subject 3	23.0	175.0	62.50
Subject 4	22.0	174.5	61.75
Average	22.75	172.5	62.29

**Table 2:** The NRMSE, *R*, and $R^{2}$ of the joint torque prediction using LSTM model.

Subject	Hip			Knee			Ankle		
	**NRMSE (%)**	R	$R^{2}$	**NRMSE (%)**	R	$R^{2}$	**NRMSE (%)**	R	$R^{2}$
Subject 1	11.7438	0.9208	0.8299	12.3561	0.9102	0.8207	5.4290	0.9696	0.9318
Subject 2	12.3223	0.9416	0.8583	14.3956	0.8973	0.7877	7.0349	0.9517	0.9010
Subject 3	14.7159	0.9201	0.7949	14.1828	0.8360	0.7949	7.6790	0.9837	0.9646
Subject 4	11.2691	0.9469	0.8512	16.8513	0.8615	0.6206	6.1971	0.9793	0.9469
Average	12.5128	0.9324	0.8336	14.4465	0.8763	0.7560	6.5850	0.9711	0.9361

**Table 3:** The NRMSE, R, and $R^{2}$ of the joint torque prediction using GPR model.

Subject	Hip			Knee			Ankle		
	**NRMSE (%)**	***R***	$R^{2}$	**NRMSE (%)**	***R***	$R^{2}$	**NRMSE (%)**	***R***	$R^{2}$
subject 1	8.6833	0.9661	0.9070	12.0694	0.9137	0.8290	5.3030	0.9558	0.8976
subject 2	14.3114	0.9165	0.8088	15.9334	0.8870	0.7400	7.7864	0.9443	0.8175
subject 3	16.1742	0.9245	0.7522	11.4735	0.9328	0.7999	6.2121	0.9801	0.9590
subject 4	10.1146	0.9491	0.8802	17.9601	0.8583	0.5690	6.6049	0.9743	0.9334
average	12.3216	0.9391	0.8371	14.3591	0.8980	0.7335	6.4766	0.9636	0.9018

## Data Availability

The data presented in this study are available on request from the corresponding author.

## References

[1] Burnfield J.M., Josephson K.R., Powers C.M., Rubenstein L.Z. (2000). The influence of lower extremity joint torque on gait characteristics in elderly men. Arch. Phys. Med. Rehabil..

[2] Kerrigan D.C., Lelas J.L., Goggins J., Merriman G.J., Kaplan R.J., Felson D.T. (2002). Effectiveness of a lateral-wedge insole on knee varus torque in patients with knee osteoarthritis. Arch. Phys. Med. Rehabil..

[3] Guo Q., Chen Z., Yan Y., Xiong W., Jiang D., Shi Y. (2022). Model identification and human-robot coupling control of lower limb exoskeleton with biogeography-based learning particle swarm optimization. Int. J. Control Autom. Syst..

[4] Chen Z., Guo Q., Li T., Yan Y., Jiang D. (2022). Gait prediction and variable admittance control for lower limb exoskeleton with measurement delay and extended-state-observer. IEEE Trans. Neural Netw. Learn. Syst..

[5] Chen Z., Guo Q., Li T., Yan Y. (2023). Output Constrained Control of Lower Limb Exoskeleton Based on Knee Motion Probabilistic Model With Finite-Time Extended State Observer. IEEE/ASME Trans. Mechatron..

[6] Gui K., Liu H., Zhang D. (2019). A practical and adaptive method to achieve EMG-based torque estimation for a robotic exoskeleton. IEEE/ASME Trans. Mechatron..

[7] Winter D.A. (2009). Biomechanics and Motor Control of Human Movement.

[8] Zajac F.E., Neptune R.R., Kautz S.A. (2002). Biomechanics and muscle coordination of human walking: Part I: Introduction to concepts, power transfer, dynamics and simulations. Gait Posture.

[9] Hill A.V. (1938). The heat of shortening and the dynamic constants of muscle. Proc. R. Soc. Lond. Ser. B-Biol. Sci..

[10] Lloyd D.G., Besier T.F. (2003). An EMG-driven musculoskeletal model to estimate muscle forces and knee joint moments in vivo. J. Biomech..

[11] Buchanan T.S., Lloyd D.G., Manal K., Besier T.F. (2004). Neuromusculoskeletal modeling: Estimation of muscle forces and joint moments and movements from measurements of neural command. J. Appl. Biomech..

[12] Lu L., Huang C., Song X. (2023). Bifurcation control of a fractional-order PD control strategy for a delayed fractional-order prey–predator system. Eur. Phys. J. Plus.

[13] Xu C., Liu Z., Li P., Yan J., Yao L. (2022). Bifurcation mechanism for fractional-order three-triangle multi-delayed neural networks. Neural Process. Lett..

[14] Li P., Peng X., Xu C., Han L., Shi S. (2023). Novel extended mixed controller design for bifurcation control of fractional-order Myc/E2F/miR-17-92 network model concerning delay. Math. Methods Appl. Sci..

[15] Shi Y., Dong W., Lin W., He L., Wang X., Li P., Gao Y. (2022). Human Joint Torque Estimation Based on Mechanomyography for Upper Extremity Exosuit. Electronics.

[16] Al-Timemy A.H., Zonnino A., Sergi F. (2020). Estimating wrist joint torque using regression ensemble of bagged trees under multiple wrist postures. Proceedings of the 2020 8th IEEE RAS/EMBS International Conference for Biomedical Robotics and Biomechatronics (BioRob).

[17] Molinaro D.D., Kang I., Camargo J., Young A.J. (2020). Biological hip torque estimation using a robotic hip exoskeleton. Proceedings of the 2020 8th IEEE RAS/EMBS International Conference for Biomedical Robotics and Biomechatronics (BioRob).

[18] Song Q., Sun B., Lei J., Gao Z., Yu Y., Liu M., Ge Y. (2006). Prediction of human elbow torque from EMG using SVM based on AWR information acquisition platform. Proceedings of the 2006 IEEE International Conference on Information Acquisition.

[19] Anwar T., Al Jumaily A. (2016). EMG signal based knee joint torque estimation. Proceedings of the 2016 International Conference on Systems in Medicine and Biology (ICSMB).

[20] Wu G., Zhang J., Li G., Wang L., Yu Q., Guo J. (2022). Identification method of nonlinear maneuver model for unmanned surface vehicle from sea trial data based on support vector machine. J. Mech. Sci. Technol..

[21] Zhang L., Li Z., Hu Y., Smith C., Farewik E.M.G., Wang R. (2020). Ankle joint torque estimation using an EMG-driven neuromusculoskeletal model and an artificial neural network model. IEEE Trans. Autom. Sci. Eng..

[22] Peng L., Hou Z.G., Wang W. (2015). A dynamic EMG-torque model of elbow based on neural networks. Proceedings of the 2015 37th Annual International Conference of the IEEE Engineering in Medicine and Biology Society (EMBC).

[23] Zhang Y., Zhang X., Lu Z., Jiang Z., Zhang T. (2020). A novel wrist joint torque prediction method based on EMG and LSTM. Proceedings of the 2020 10th Institute of Electrical and Electronics Engineers International Conference on Cyber Technology in Automation, Control, and Intelligent Systems (CYBER).

[24] Dai X., Andani H.T., Alizadeh A., Abed A.M., Smaisim G.F., Hadrawi S.K., Karimi M., Shamsborhan M., Toghraie D. (2023). Using Gaussian Process Regression (GPR) models with the Matérn covariance function to predict the dynamic viscosity and torque of SiO2/Ethylene glycol nanofluid: A machine learning approach. Eng. Appl. Artif. Intell..

[25] Nguyen-Tuong D., Seeger M., Peters J. (2008). Computed torque control with nonparametric regression models. Proceedings of the 2008 American Control Conference.

[26] Pei X., Zhou Y., Wang N. (2019). A Gaussian process regression based on variable parameters fuzzy dominance genetic algorithm for B-TFPMM torque estimation. Neurocomputing.

[27] Siu H.C., Sloboda J., McKindles R.J., Stirling L.A. (2021). A neural network estimation of ankle torques from electromyography and accelerometry. IEEE Trans. Neural Syst. Rehabil. Eng..

[28] Guo Q., Zhang Y., Celler B.G., Su S.W. (2019). Neural adaptive backstepping control of a robotic manipulator with prescribed performance constraint. IEEE Trans. Neural Netw. Learn. Syst..

[29] Guo Q., Chen Z. (2021). Neural adaptive control of single-rod electrohydraulic system with lumped uncertainty. Mech. Syst. Signal Proc..

[30] Zhang L., Soselia D., Wang R., Gutierrez-Farewik E.M. (2022). Lower-limb joint torque prediction using LSTM neural networks and transfer learning. IEEE Trans. Neural Syst. Rehabil. Eng..

[31] Truong M.T.N., Ali A.E.A., Owaki D., Hayashibe M. (2023). EMG-Based Estimation of Lower Limb Joint Angles and Moments Using Long Short-Term Memory Network. Sensors.

[32] Yang J., Yin Y. (2020). Dependent-Gaussian-process-based learning of joint torques using wearable smart shoes for exoskeleton. Sensors.

[33] Ullauri J.B., Peternel L., Ugurlu B., Yamada Y., Morimoto J. (2015). On the EMG-based torque estimation for humans coupled with a force-controlled elbow exoskeleton. Proceedings of the 2015 International Conference on Advanced Robotics (ICAR).

[34] Wang M., Chen Z., Zhan H., Zhang J., Wu X., Jiang D., Guo Q. (2023). Lower limb joint torque estimation by neural network and Sparse Gaussian Process with RIO Kernel. Proceedings of the 2008 8th International Conference on Advanced Robotics and Mechatronics.

[35] Moreira L., Figueiredo J., Fonseca P., Vilas-Boas J.P., Santos C.P. (2021). Lower limb kinematic, kinetic, and EMG data from young healthy humans during walking at controlled speeds. Sci. Data.

[36] Li G., Li J., Ju Z., Sun Y., Kong J. (2019). A novel feature extraction method for machine learning based on surface electromyography from healthy brain. Neural Comput. Appl..

[37] Toledo-Perez D., Rodriguez-Resendiz J., Gomez-Loenzo R.A. (2020). A study of computing zero crossing methods and an improved proposal for EMG signals. IEEE Access.

[38] Bhattacharya A., Sarkar A., Basak P. (2017). Time domain multi-feature extraction and classification of human hand movements using surface EMG. Proceedings of the 2017 4th International Conference on Advanced Computing and Communication Systems (ICACCS).

[39] Thongpanja S., Phinyomark A., Phukpattaranont P., Limsakul C. (2013). Mean and median frequency of EMG signal to determine muscle force based on time-dependent power spectrum. Elektron. Elektrotechnika.

[40] Hochreiter S., Schmidhuber J. LSTM can solve hard long time lag problems. Proceedings of the 9th International Conference on Neural Information Processing Systems.

[41] Gers F.A., Schmidhuber J., Cummins F. (2000). Learning to forget: Continual prediction with LSTM. Neural Comput..

[42] Cao D., Zhao J., Hu W., Zhang Y., Liao Q., Chen Z., Blaabjerg F. (2021). Robust deep Gaussian process-based probabilistic electrical load forecasting against anomalous events. IEEE Trans. Ind. Inform..

[43] Qiu X., Meyerson E., Miikkulainen R. (2019). Quantifying point-prediction uncertainty in neural networks via residual estimation with an i/o kernel. arXiv.

[44] Titsias M. Variational learning of inducing variables in sparse Gaussian processes. Proceedings of the Artificial Intelligence and Statistics, PMLR.

[45] Rasmussen C.E., Williams C.K. (2006). Gaussian Processes for Machine Learning.

[46] Koriyama T., Kobayashi T. (2019). Statistical parametric speech synthesis using deep Gaussian processes. IEEE/ACM Trans. Audio Speech Lang. Process..

